# Design and field evaluation of a lateral flow cassette device for point-of-care bilirubin measurement

**DOI:** 10.1371/journal.pgph.0002262

**Published:** 2023-08-08

**Authors:** Alyssa Shapiro, Prince Mtenthaonga, Rowland Mjumira, Margaret Reuben, Ayodele Samuel, Meaghan Bond, Jennifer Carns, Richard Schwarz, Ryan Johnston, Lucky Mangwiro, Opeyemi Odedere, Robert Miros, Sean McHugh, Kondwani Kawaza, Queen Dube, Chinyere Ezeaka, Rebecca Richards-Kortum

**Affiliations:** 1 Department of Bioengineering, Rice University, Houston, Texas, United States of America; 2 Rice360 Institute for Global Health Technologies, Rice University, Houston, Texas, United States of America; 3 Department of Pediatrics, Queen Elizabeth Central Hospital, Blantyre, Malawi; 4 Department of Paediatrics, College of Medicine, University of Lagos, Lagos, Nigeria; 5 Kamuzu University of Health Sciences, Blantyre, Malawi; 6 3rd Stone Design, San Rafael, California, United States of America; 7 DCN Dx, Carlsbad, California, United States of America; Indian Institute of Technology Bombay, INDIA

## Abstract

Neonatal jaundice is an important cause of morbidity and mortality worldwide, and neonates born in low and middle-income countries bear a disproportionate burden. We previously developed a low-cost, point-of-care system to measure total serum bilirubin (TSB) in neonates. This device was effective at detecting and monitoring jaundice; however, the disposable strips were difficult to produce at scale. Here, we report a new lateral flow cassette design, called BiliDx, that was produced at scale using traditional manufacturing techniques. We evaluated the performance of BiliDx at sites in Nigeria and Malawi. The lateral flow strip consists of plasma separation membranes, nitrocellulose, and a plastic cassette. We evaluated the performance of the strips and reader at two hospitals located in Nigeria and Malawi compared to reference standard TSB. We also assessed performance for samples with high direct bilirubin (DB) and high hematocrit (HCT). We collected 1,144 samples from 758 neonates (TSB ranged from 0.2 to 45.9 mg/dL). The mean bias of BiliDx measurements in the validation set was +0.75 mg/dL, and 95% limits of agreement were -2.57 to 4.07 mg/dL. The mean bias and limits of agreement were comparable for samples with HCT < 60% and HCT ≥ 60%, and for samples with low and intermediate DB levels; the samples with high DB levels had wider 95% limits of agreement (-4.50 to +3.03 mg/dL). Error grid analysis shows that 96.9% of samples measured with BiliDx would have resulted in the same clinical decision as the reference standard. This performance is comparable to previous results that used a handmade two-dimensional strip. Additionally, error grid analysis shows that all 20 samples with high DB levels would have resulted in the same clinical decision as the reference standard. This evaluation supports the use of BiliDx lateral flow cassettes to provide accurate point-of-care measurements in low-resource settings.

## Introduction

Neonatal jaundice is an important cause of neonatal morbidity and mortality worldwide [1]. More than 80% of newborns develop jaundice [2,3], which is usually benign if detected and treated in a timely manner [4]. However, 10% of term and 25% of preterm babies develop significantly elevated serum bilirubin levels that require treatment [5]. Failure to provide bilirubin estimation and prompt treatment places these neonates at risk of severe brain damage and death [6]. Low and middle-income countries (LMICs) bear a disproportionate burden of jaundice-related mortality and morbidity; the burden is highest in South Asia and sub-Saharan Africa, where jaundice is the 7^th^ and 8^th^ leading cause of neonatal mortality, respectively [1]. In the early neonatal period (0–6 days), neonatal jaundice accounted for 1,954 deaths per 100,000 live births in sub-Saharan Africa, compared to just 18.4 deaths per 100,000 live births in North America [1].

Neonatal jaundice is easily treated using phototherapy, a technology that photodecomposes bilirubin into a form that can be easily excreted from the body. Many promising low-cost phototherapy lights have emerged in recent years, including LED-based phototherapy machines [7]. However, there is still a need for affordable, reliable, point-of-care (POC) bilirubin measurement tools which can identify jaundiced neonates and guide phototherapy treatment in low-resource settings.

Existing technologies for bilirubin measurement are prohibitively expensive for LMICs and/or are not sufficiently accurate. Standard-of-care laboratory methods to measure total serum bilirubin (TSB) include high-performance liquid chromatography, diazo reactions, and spectrophotometric benchtop bilirubinometers; these techniques require expensive equipment often unavailable in LMICs [8,9]. Many hospitals in LMICs without access to laboratory TSB measurement rely on visual inspection of the skin and sclera, such as with Kramer’s method [8,10,11]; however, visual inspection alone, while useful for ruling out jaundice, is inaccurate for assessing the severity of jaundice and estimating bilirubin levels [12–14]. Transcutaneous bilirubinometry (TcB), a noninvasive bilirubin measurement through the skin, is recommended for use primarily as a screening tool to determine whether TSB should be measured [15–17]. In LMICs, TcB has been used as a replacement for TSB when TSB is unavailable instead of as a screening tool [8,10,18]; however, TcB accuracy has been shown to be population-dependent and can be especially inaccurate for neonates with darker skin tones [19–21]. Serum-based TSB measurement methods must have acceptable accuracy for samples with high hematocrit (HCT), because neonatal HCT is 53% on average and varies greatly with GA and chronological age, among other factors [22]. Samples with higher HCT also have less plasma available for TSB measurement; thus, obtaining sufficient plasma for measurement is a challenge for any plasma-based measurement tool. Several point-of-care TSB measurement devices have limited accuracy at high HCT levels [23–26]. Some point-of-care TSB methods are commercially available [24,27–29] and some are under development [30–32]; however, there is still a need in LMICs for an accurate rapid, affordable, point-of-care bilirubin test.

We previously developed BiliSpec, a low-cost point-of-care system to measure TSB in neonates. The BiliSpec system consists of a handmade paper-based strip, which separates plasma from capillary blood collected from a heelstick, and a handheld spectrophotometric reader. We evaluated the accuracy of BiliSpec compared to gold standard TSB in a pilot study involving 68 neonates [33] and in a validation study involving 375 neonates in two hospitals in Malawi [34]. While BiliSpec performed well compared to a reference standard in both studies, the handmade strip design was difficult to produce at scale. Additionally, in our previous work, we encountered several neonates for which BiliSpec consistently underestimated TSB; we suspected this difference could be due to the presence of direct bilirubin (DB). This is because the molar absorption coefficient of direct bilirubin is lower at the wavelength measured by BiliSpec (470 nm) than the wavelength measured by the reference standard (460 nm), but the molar absorption coefficient of indirect (unconjugated) bilirubin is comparable at the two wavelengths [35]. This difference could theoretically cause BiliSpec to underestimate bilirubin in samples with higher quantities of direct bilirubin. In our previous study, laboratory methods to determine DB levels were not available to investigate this hypothesis.

In this work, we designed and evaluated a lateral flow cassette that can be produced at scale using traditional manufacturing techniques. This cassette, along with the previously developed reader, are together referred to as BiliDx. In this study, we evaluated the performance of BiliDx at two hospitals located in Nigeria and Malawi compared to gold standard TSB methods and to TcB. We collected 1144 samples from 758 neonates with TSB concentrations ranging from 0.2 to 45.9 mg/dL. We assessed BiliDx accuracy at high HCT levels and in samples with high direct bilirubin (DB). Finally, we considered the potential clinical impact of bilirubin measurement errors using bilirubin error grids, developed previously [34] and now updated based on the American Academy of Pediatrics (AAP) 2022 guidelines [36].

## Methods

### Ethics statement

Hospitalized neonates whose guardians gave informed consent were eligible to participate in the study. Written informed consent was obtained from the parent/guardian of each participant. The study was reviewed and approved by the Malawi College of Medicine Research Ethics Committee (COMREC 2435), the Lagos University Teaching Hospital Health Research Ethics Committee (NHREC 19/12/2008a), and the Rice University Institutional Review Board (IRB-FY2018-463).

### BiliDx cassette design

**Fig 1:**
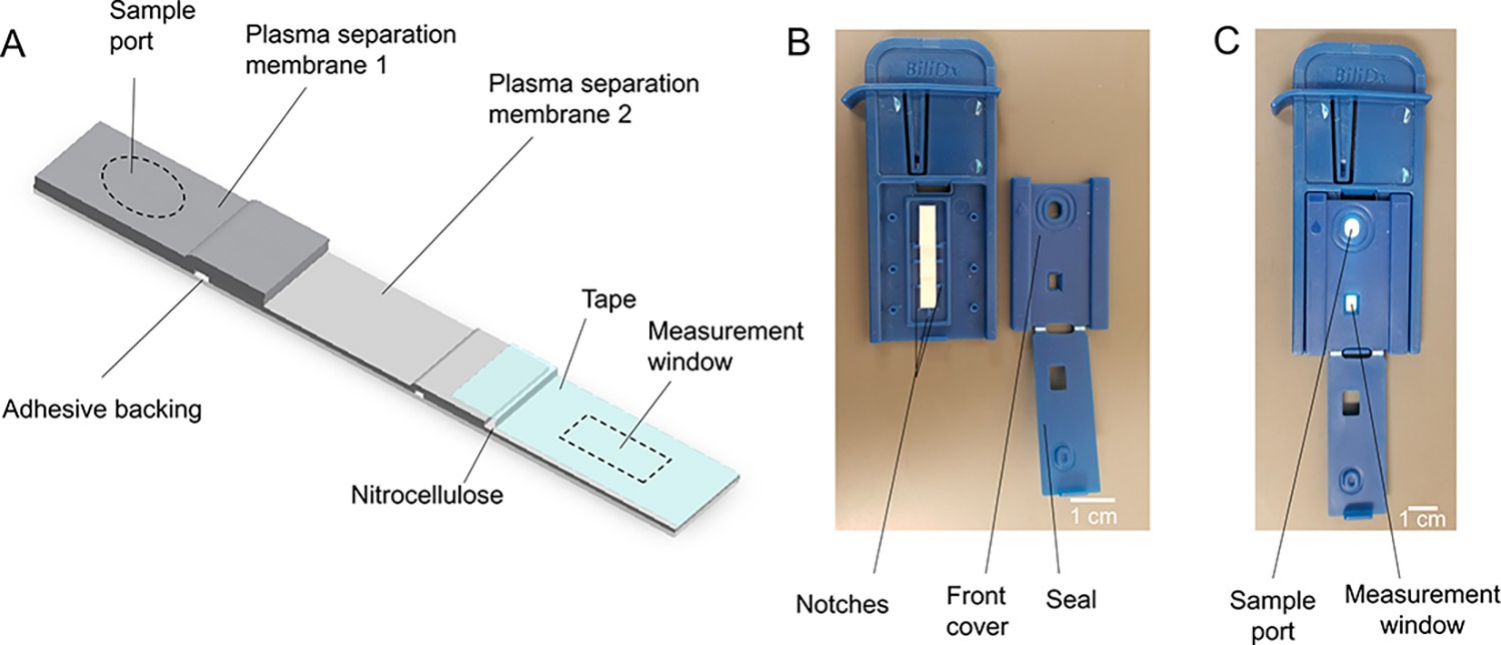
BiliDx cassette design. A) Diagram of the lateral flow strip components, placed atop a plastic adhesive backing. B) The lateral flow strip is enclosed in an injection molded plastic cassette. Notches in the plastic cassette ensure consistent alignment of the lateral flow strip for measurement. C) Fully assembled cassette. Whole blood is added to the sample port; plasma is separated from whole blood and flows towards the measurement window, where bilirubin measurement takes place.

The BiliDx cassette consists of a lateral flow strip inside a plastic housing. The lateral flow strip (Fig 1A) contains two plasma separation membranes: Whatman VF2 and Whatman LF1 glass fiber. Separated plasma wicks onto a Sartorius CN95 nitrocellulose sheet with clear plastic backing, covered with Lohmann GL-163 tape. The strip components are assembled onto an adhesive backing. The one-dimensional strip is manufactured at scale using traditional lateral flow manufacturing techniques. The strips are placed inside an injection-molded plastic housing (Fig 1B). The plastic housing contains notches to align the lateral flow strip relative to a measurement window. The front cover seals using a living hinge which snaps over the top of the front cover after blood is applied. The fully assembled plastic cassette (Fig 1C) contains two openings: a circular sample port above the first plasma separation membrane where blood is applied, and a rectangular measurement window above the nitrocellulose where the sample is measured by the BiliDx reader.

### BiliDx workflow

**Fig 2:**
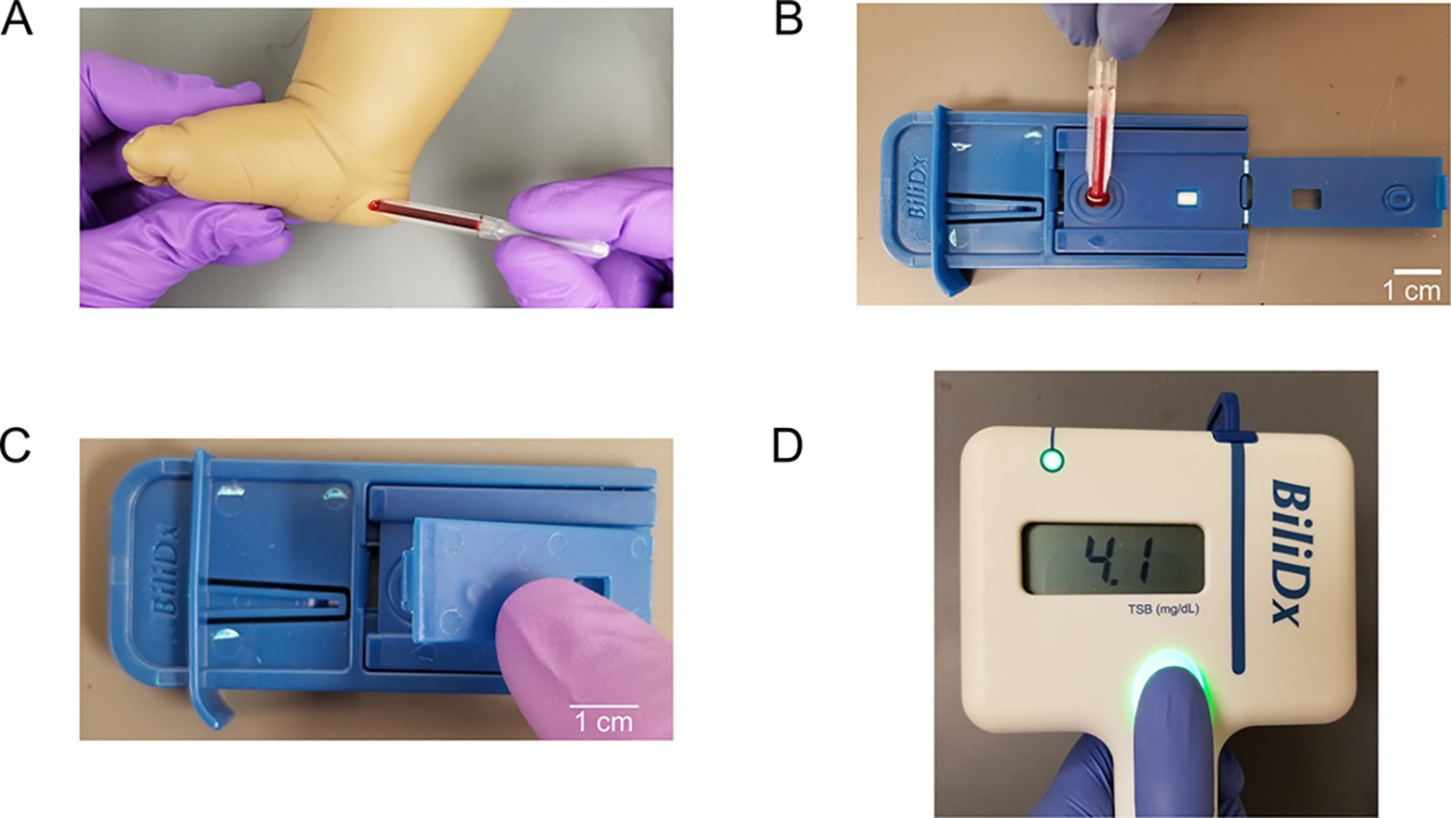
BiliDx workflow. A) A heel stick is performed on a neonate, and capillary blood is collected into a 75 μL plastic transfer pipette. B) Blood is applied to the sample port of the BiliDx cassette. C) The user folds the seal over the sample port to close the cassette. D) The strip is then inserted into the BiliDx device. The user pushes the measurement button, and a TSB reading appears on the screen in a few seconds.

Using a MICROSAFE capillary tube, 75 μL of capillary blood from a heelprick (Fig 2A) is applied to the sample port (Fig 2B), and the front cover of the plastic housing is sealed (Fig 2C). Once the cassette window is visibly filled with plasma (~2–10 minutes), the cassette is inserted into the reader (Fig 2D). A few seconds after pressing the measurement button, the measured value of total serum bilirubin (TSB) is displayed on the screen.

The BiliDx reader has been described previously [33,34]. Briefly, the reader measures light transmission through plasma on nitrocellulose. Optical density (OD) is measured and calculated in three spectral regions (peak wavelengths at 470 nm, 590 nm, and 660 nm) [37], which account for absorption due to bilirubin and hemoglobin as well as background absorbance due to nitrocellulose [34]. The reader required a simple update to the algorithm to adjust for new cassette parameters. OD values are also used to detect whether an inserted strip is filled with plasma; an error message is displayed to indicate when strips are underfilled. The user can either measure the cassette again once plasma completely fills the measurement window, or he/she can collect another sample and apply it to a new cassette.

### Clinical evaluation of BiliDx

A clinical study was conducted at two central hospitals: Queen Elizabeth Central Hospital (QECH) in Blantyre, Malawi and Lagos University Teaching Hospital (LUTH) in Lagos, Nigeria to: 1) update the algorithm of the BiliDx reader for TSB measurement with the new lateral flow cassette design and 2) assess the accuracy of the optimized BiliDx system relative to a reference laboratory standard. Accuracy was assessed across a range of hematocrit (HCT) and direct bilirubin (DB) levels. Transcutaneous bilirubin (TcB) values were also measured and accuracy was assessed using the reference laboratory standard.

Patients were enrolled from June 10, 2020 –December 1, 2021. Date of birth, birth weight, gestational age (GA), gender, comorbidities, and other relevant medical history were recorded for each study participant. When gestational age was unknown, it was estimated retrospectively based on birth weight [34].

Bilirubin measurements were obtained throughout hospitalization as directed by the treating clinician, based on a variety of clinical factors including laboratory standard TSB measurements; neither BiliDx nor TcB measurements were used to make clinical decisions. Research personnel measured TcB using a JM-105 transcutaneous bilirubinometer (Draeger). Five measurements were taken on the forehead; the average of the measurements was recorded. TcB accurately measures bilirubin concentration for areas of skin not exposed to phototherapy treatment [38]. Thus, if the neonate was receiving phototherapy treatment, a protective eye cover was used to shield the forehead during treatment administration, and TcB measurements were measured from a shielded region of the forehead. Then, study personnel performed a heel prick on the neonate to obtain capillary blood. First, 75 μL of capillary blood was collected from the heel using a MICROSAFE plastic capillary tube and applied to the sample port of a BiliDx cassette. The same cassette was measured using two BiliDx devices (in the study at LUTH) or using three BiliDx devices (in the study at QECH).

For samples collected from June 2020 –May 2021, BiliDx devices were calibrated daily using three neutral density filters and one unused BiliDx cassette; the daily calibration values were analyzed to determine optimal frequency of calibration. We determined that optimal calibration frequency was once at the factory using neutral density filters, and every 25 measurements by users with an unused BiliDx cassette; this recommended calibration frequency will be communicated to BiliDx users via a user manual and prompted by the device itself. For samples collected from May 2021 –December 2021, BiliDx was calibrated once with neutral density filters (in May 2021) and once every 25 measurements with an unused BiliDx cassette.

A few drops of additional capillary blood were collected into an EDTA Microtainer tube (BD, Catalog # 365974) for reference standard TSB measurement and HCT measurement. Blood was collected from the Microtainer tube via capillary tube for HCT measurement using a ZIP Combo Centrifuge (LW Scientific). Then, the Microtainer tube was centrifuged to separate plasma. At both sites, 20 μL of plasma was measured using a UNISTAT bilirubinometer to obtain TSB (Reichert Technologies). Additionally, at one site (LUTH), 30 μL of plasma was measured using an Advanced BR2 Bilirubin Stat-Analyzer bilirubinometer (Advanced Instruments) to obtain both TSB and direct bilirubin (DB) levels. TSB values measured with the UNISTAT and BR2 devices correlated well (r = 0.996); TSB measured with the UNISTAT was used as the reference standard to evaluate the accuracy of both BiliDx and TcB since a UNISTAT was available at both sites. For those samples measured with the BR2 device, the percentage of direct bilirubin was calculated as DB divided by TSB, as measured with the BR2. Samples with high fractions of direct bilirubin were defined as samples with DB levels of either >2 mg/dL or >20% of TSB, in accordance with published guidelines [39,40].

The UNISTAT, BR2, and TcB bilirubinometers were calibrated according to manufacturer’s specifications. Additionally, to ensure consistency across sites, the two UNISTAT devices were calibrated to agree with a third reference UNISTAT bilirubinometer, located in the Malaria Research Ward in QECH [34].

We selected a sample size with the goal of ensuring collection of a sufficient number of samples with TSB concentration > 20 mg/dL to ensure that the device performs well at high TSB levels. In our previous study [34], we collected 475 samples, of which 26 had a TSB value above 20 mg/dL. We also found a standard deviation between BiliDx and the reference standard of 2.2 mg/dL. In order to detect a statistically significant difference between results measured by the Bilirubinometer and our device of 1.8 mg/dL (power = 80%, α = 0.05) using a paired test [41], 25 samples are required with a TSB > 20mg/dL. We thus aimed to collect 25 samples with TSB values > 20 mg/dL in both the training and validation sets during this study. Based on our previous study, this would require 913 samples; to account for possible sample loss or variability in the distribution of bilirubin concentrations encountered, we increased this sample size to approximately 1100.

### Data analysis

BiliDx cassettes were analyzed automatically by the BiliDx reader to determine whether they were adequately filled with plasma. BiliDx displayed an error message to the user if the strip was unfilled; measurements that generated this error message were excluded from analysis.

Data collected from all BiliDx readers from the first 655 samples entered into the database were used to update a previously determined bilirubin prediction algorithm [34] (training set). Data collected from the remaining 446 samples were used to evaluate the performance of the algorithm (validation set). Validation set samples were stratified by HCT and DB levels to assess impact on accuracy.

To evaluate the potential clinical impact of bilirubin measurement errors, data were plotted on bilirubin measurement error grids [34]. We previously developed bilirubin error grids [34], inspired by Clarke error grids for glucose concentration measurement [42], to visualize and evaluate clinical significance of TSB measurement errors based on guidelines for phototherapy and exchange transfusion. In this paper, we have updated the error grids to reflect 2022 AAP guidelines for initiation of phototherapy and exchange transfusion for infants at 35 weeks gestation or older [36]. Horizontal and vertical red lines shown on each grid indicate thresholds for phototherapy and exchange transfusion treatment; these thresholds vary based on gestational age at birth, day of life, and the neonate’s risk level for jaundice, according to applicable guidelines [36,43]. The proposed Clinical Laboratory Improvement Amendments (CLIA) recommendations for bilirubin laboratory measurement accuracy are shown as solid black lines at ±20% of the reference standard [44]. Regions in the grids are color-coded from green to red, indicating increasing clinical impact of bilirubin measurement error. A measurement that falls in a green region is either within the proposed CLIA guidelines or would result in the correct clinical decision (e.g., Region A: phototherapy not clinically required or indicated). A measurement that falls in a red region indicates under-treatment potentially resulting in significant clinical harm (e.g., Region E: exchange transfusion clinically required but no treatment indicated). Data in the validation set were plotted on one of fifty-eight error grids based on the gestational age at birth, day of life, and risk level for jaundice, according to applicable guidelines [36,43]. Samples for which the TcB reported a non-numeric “>20” were not included in error grid analysis.

## Results

**Fig 3:**
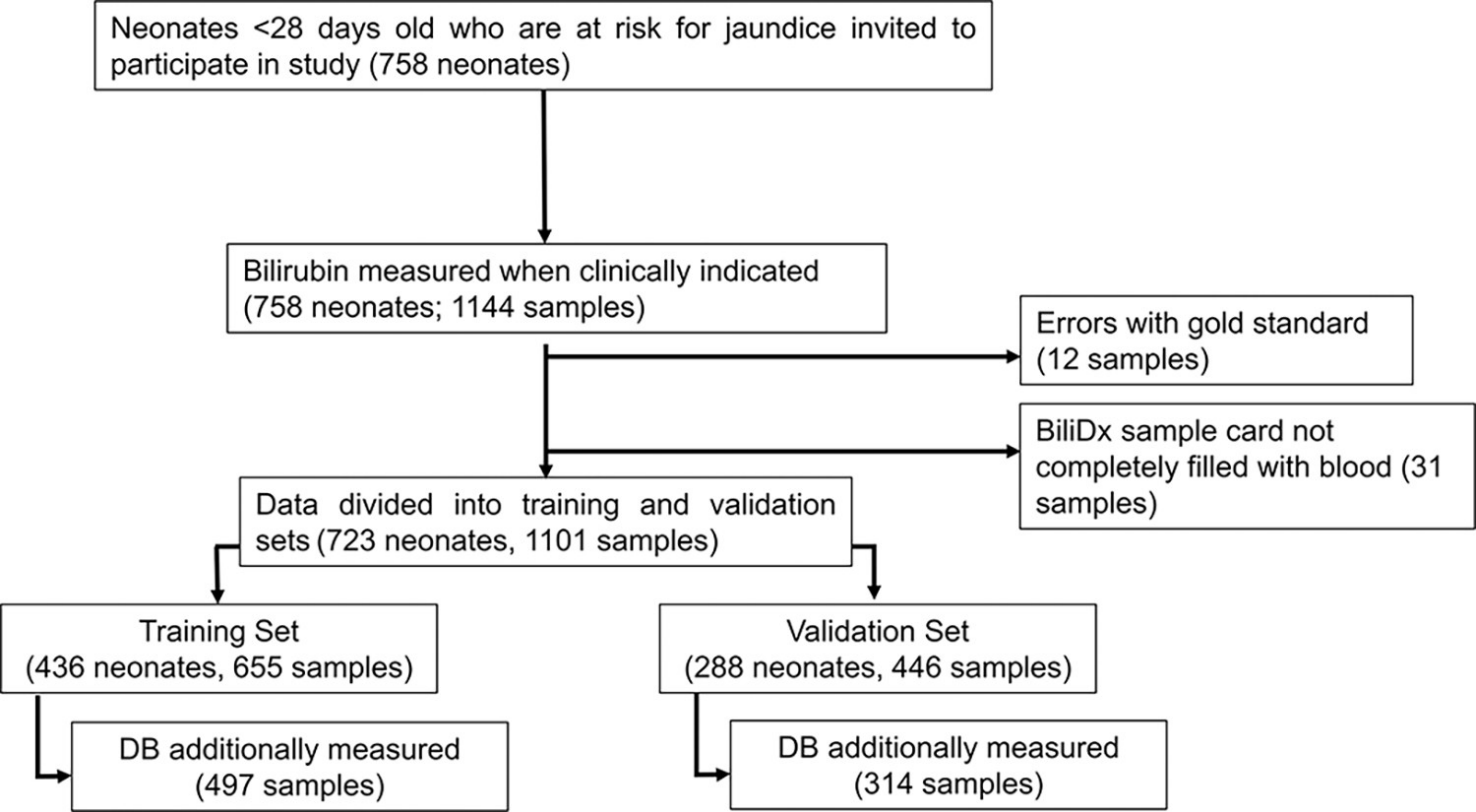
Study schematic. Samples were divided into a training set (first 655 samples entered into the database) and validation set (remaining 446 samples). For one patient, one sample was included in the training set and one sample was included the validation set. Data from the training set were used to update the BiliDx algorithm for the new cassette. Data from the validation set were used to evaluate the performance of BiliDx with the new algorithm. Direct bilirubin (DB) was additionally measured in a subset of samples in both the training and validation sets.

We enrolled 758 neonates from whom 1144 samples were collected (Fig 3). Twelve samples (1.1%) were excluded due to errors with the UNISTAT reference standard, such as missing measurements, under-filled UNISTAT cuvettes, or visible red blood cells or debris in cuvettes. Thirty-one samples (2.7%) were excluded because the BiliDx cassette did not adequately fill with plasma and the BiliDx device displayed an error message to the user. The remaining 1101 samples from 723 neonates were divided into training (655 samples) and validation (446 samples) by date collected. For a subset of samples, both TSB and DB levels were measured (497 samples in the training set and 314 samples in the validation set).

Patient and sample characteristics are shown in Table 1. Three hundred ninety-four (54.5%) patients were male. Six hundred sixty-seven (60.6%) measurements were taken from a shielded region of the forehead while the neonate underwent phototherapy treatment. Estimated GA at birth varied from 24 weeks to over 35 weeks and birth weight ranged from 620 g to 5000 g. Sample hematocrit ranged from 17% to 80%; the mean was 48.8%. DB levels were measured for 811 samples; of these samples, 439 (54.1%) had little or no direct bilirubin (DB < 0.2 mg/dL), while 80 (9.9%) had high levels of direct bilirubin (DB > 2 mg/dL or > 20% of TSB) [39,40].

**Table 1:** Patient and sample characteristics for samples included in analysis.

	Total	Training Set			Validation Set		
		Total	QECH	LUTH	Total	QECH	LUTH
**Number of neonates**	**723** ^**1**^	**436**	141	295	**288**	115	173
Male neonates (% of total number of neonates from each hospital)	**394** **(54.5%)**	**239** **(54.8%)**	84 (59.6%)	155 (52.5%)	**155** **(53.8%)**	66 (57.4%)	89 (51.5%)
**Number of samples**	**1101**	**655**	155	500	**446**	132	314
Number of samples obtained when neonate undergoing phototherapy (% of total number of samples from each hospital)	**667** **(60.6%)**	**390** **(59.5%)**	127 (81.9%)	263 (52.6%)	**277** **(62.1%)**	101 (76.5%)	176 (56.1%)
Number of samples obtained when neonate had received exchange transfusion^2^	**78** **(7.1%)**	**41** **(6.3%)**	0	41 (8.2%)	**37** **(8.3%)**	0	37 (11.8%)
**Neonatal birth weight (g)**							
<1145 (GA < 28 wks)	**32**	**16**	6	10	**16**	5	11
1145 – 1404 (GA 28-30 wks)	**38**	**22**	10	12	**16**	8	8
1404-1821 (GA 30-32 wks)	**93**	**60**	26	34	**33**	20	13
1821-2201 (GA 32-34 wks)	**84**	**48**	23	25	**36**	22	14
2201 – 2394 (GA 34-35 wks)	**24**	**12**	2	10	**12**	5	7
> 2394 (GA >= 35 wks)	**402**	**244**	74	170	**159**	53	106
Not recorded	**50**	**34**	0	34	**16**	2	14
**Sample TSB (reference standard, mg/dL)**							
0 – 5	**312**	**165**	15	150	**147**	18	129
5 – 10	**463**	**291**	71	220	**172**	48	124
10 – 15	**205**	**136**	46	90	**69**	33	36
15 – 20	**72**	**41**	17	24	**31**	22	9
20 – 25	**24**	**12**	4	8	**12**	6	6
25 – 30	**13**	**5**	0	5	**8**	2	6
30 – 35	**9**	**3**	1	2	**6**	3	3
> 35	**3**	**2**	1	1	**1**	0	1
Mean Value (mg/dL)	**8.5**	**8.5**	10.2	8.0	**8.5**	11.1	7.4
Median Value (mg/dL)	**7.5**	**7.8**	9.3	7.2	**7.1**	10.0	5.9
**Sample Hematocrit**							
18% – 36%	**64**	**42**	11	31	**22**	10	12
36% – 50%	**511**	**319**	55	264	**192**	43	149
50% – 60%	**396**	**236**	61	175	**160**	46	114
60% – 70%	**100**	**45**	16	29	**55**	23	32
> 70%	**23**	**9**	9	0	**14**	7	7
Not recorded	**7**	**4**	3	1	**3**	3	0
**Samples with direct bilirubin measurement**							
0 - 0.2 mg/dL	**439**	**284**	n/a	284	**155**	n/a	155
0.2 - 2 mg/dL	**307**	**167**	n/a	167	**140**	n/a	140
2 – 4 mg/dL	**26**	**20**	n/a	20	**6**	n/a	6
4 – 6 mg/dL	**15**	**12**	n/a	12	**3**	n/a	3
> 6 mg/dL	**24**	**14**	n/a	14	**10**	n/a	10
Not available	**290**	**158**	n/a	3	**132**	n/a	0

^1^For one patient, one sample was included in the training set (first 655 samples entered into the database) and one sample was included the validation set (remaining 446 samples). QECH = Queen Elizabeth Central Hospital; LUTH = Lagos University Teaching Hospital, GA = gestational age.

^2^QECH did not perform exchange transfusions.

**Fig 4:**
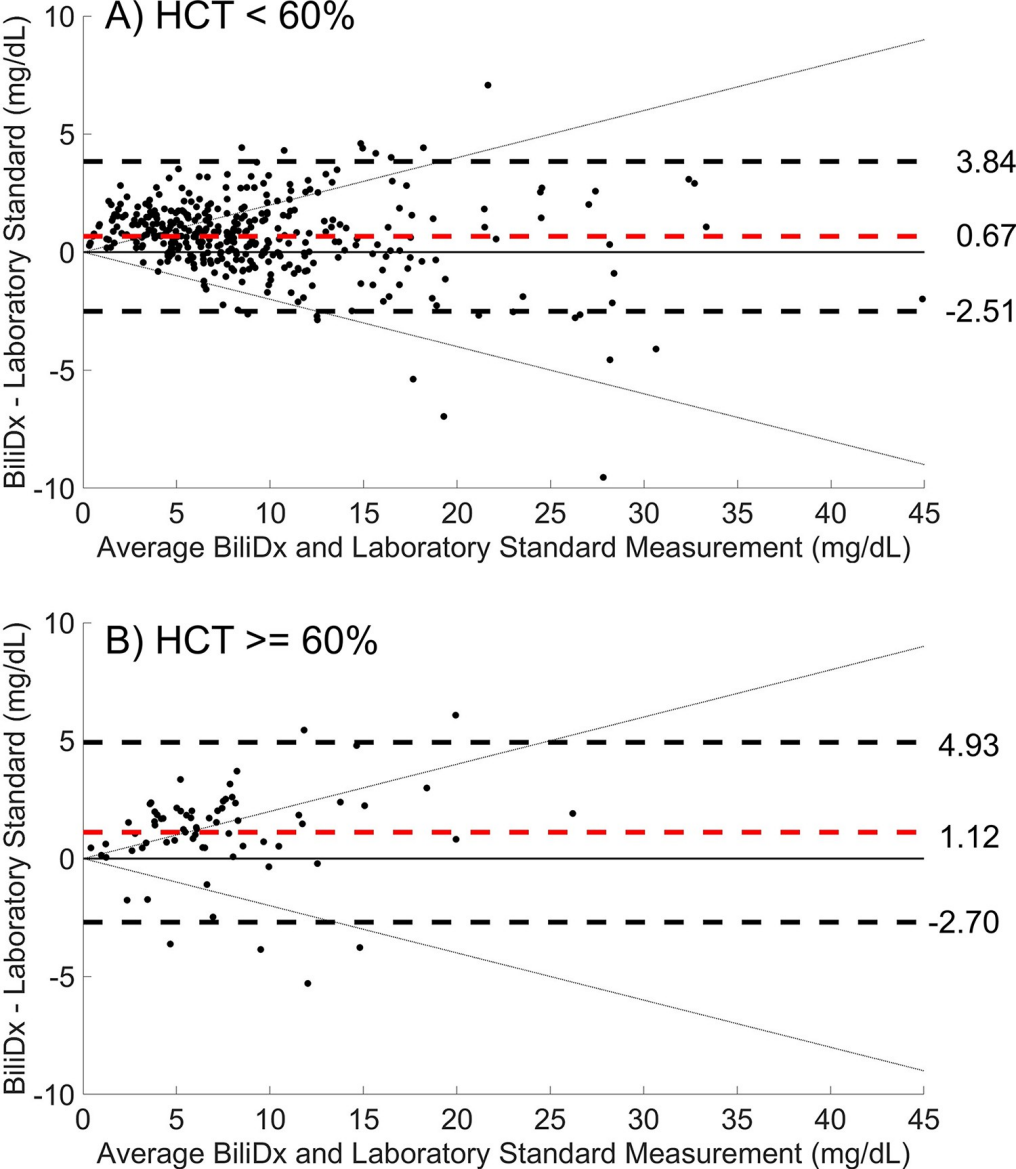
BiliDx performance for samples in the validation set, stratified by hematocrit level. Bland-Altman plots for validation set samples comparing TSB (Total Serum Bilirubin) measured using BiliDx to that measured using a reference standard (UNISTAT), stratified by hematocrit (HCT): A) HCT < 60% (374 samples), and B) HCT ≥ 60% (69 samples). Dashed red lines indicate mean bias; dashed black lines indicate 95% limits of agreement. Diagonal dotted black lines indicate CLIA proposed guidelines of ± 20%.

Fig 4 shows Bland-Altman plots for TSB values measured by BiliDx versus reference standard values (UNISTAT) for samples in the validation set, stratified by HCT level. The overall mean bias of BiliDx measurements in the validation set was + 0.75 mg/dL, and 95% limits of agreement were -2.57 to 4.07 mg/dL; the mean bias and 95% limits of agreement were comparable for samples with HCT < 60% (Fig 4A) and samples with HCT ≥ 60% (Fig 4B). Results are shown from one representative BiliDx reader per site; results from all BiliDx readers and all HCT levels are shown in S1 Fig.

Data from all BiliDx readers correlated well with each other; the Pearson correlation coefficients (r) were found to be 0.97–0.98 between pairs of devices, and 2–3 measurements of samples in the validation set by multiple BiliDx devices had a pooled standard deviation of 1.1 mg/dL.

**Fig 5:**
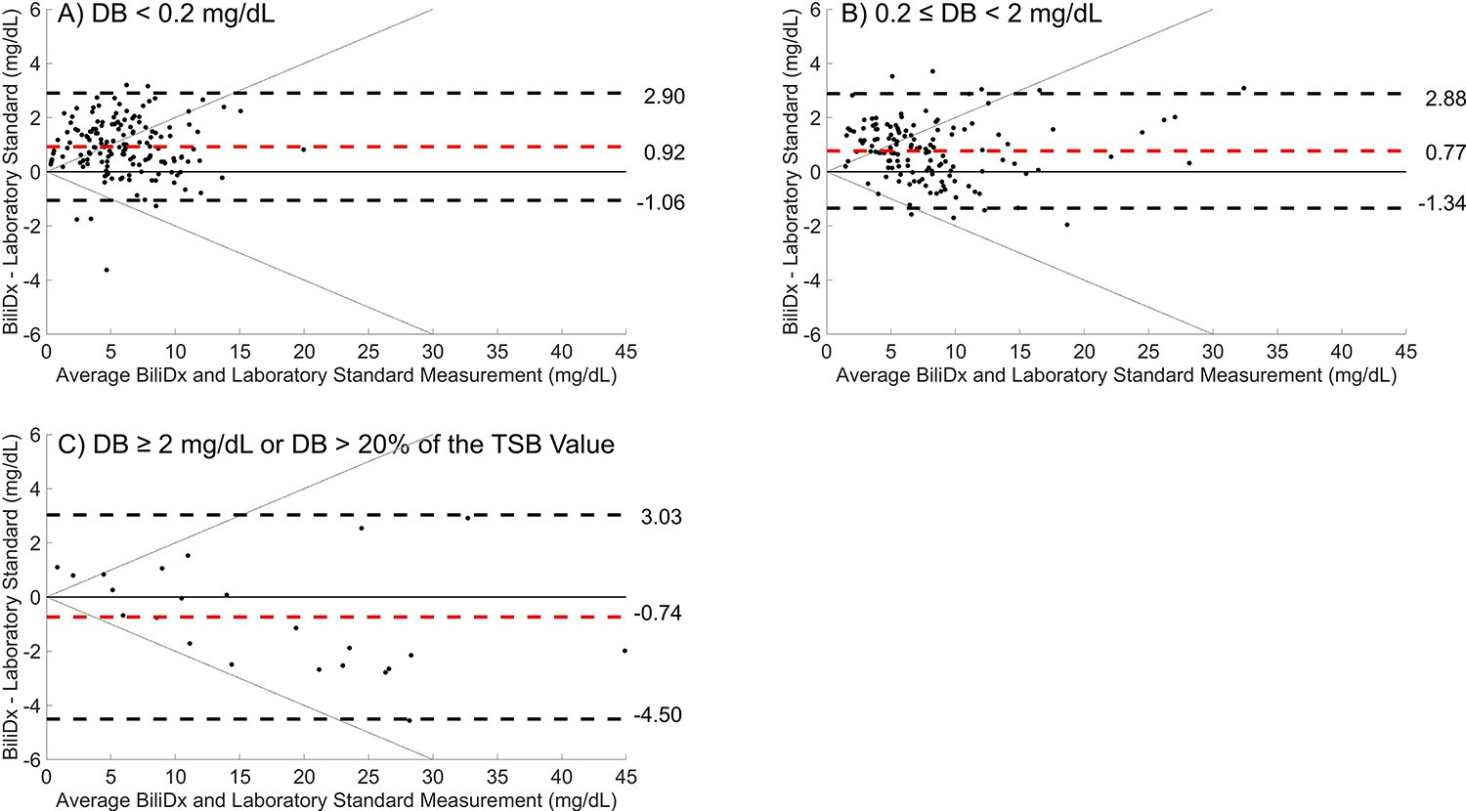
BiliDx performance for samples in the validation set, stratified by direct bilirubin (DB) levels. Bland-Altman plots for validation set samples comparing TSB (Total Serum Bilirubin) measured using BiliDx to that measured using the reference standard (UNISTAT), stratified by direct bilirubin levels as measured by the BR2 bilirubinometer. A) Samples with DB < 0.2 mg/dL (155 samples). B) Samples with DB between 0.2 and 2 mg/dL, and with DB levels < 20% of the total bilirubin levels (135 samples). C) Samples with either DB > 2 mg/dL or DB > 20% of the total bilirubin level (23 samples). Dashed red lines indicate mean bias; dashed black lines indicate 95% limits of agreement. Diagonal dotted black lines indicate CLIA proposed guidelines of ± 20%.

Fig 5 shows Bland-Altman plots for BiliDx TSB values versus the reference standard (UNISTAT) for samples in the validation set, stratified by DB levels. Samples with DB levels less than 0.2 mg/dL are shown in Fig 5A; samples with DB levels between 0.2 and 2 mg/dL and with DB levels < 20% of the total bilirubin levels are shown in Fig 5B; and samples with high DB fractions (DB > 2 mg/dL or > 20% of TSB) are shown in Fig 5C. Mean bias and 95% limits of agreement are comparable for samples with low and intermediate DB levels (Fig 5A and 5B). The samples with high DB levels have a comparable mean bias of -0.74 mg/dL but wider 95% limits of agreement of -4.50 to +3.03 mg/dL.

**Fig 6:**
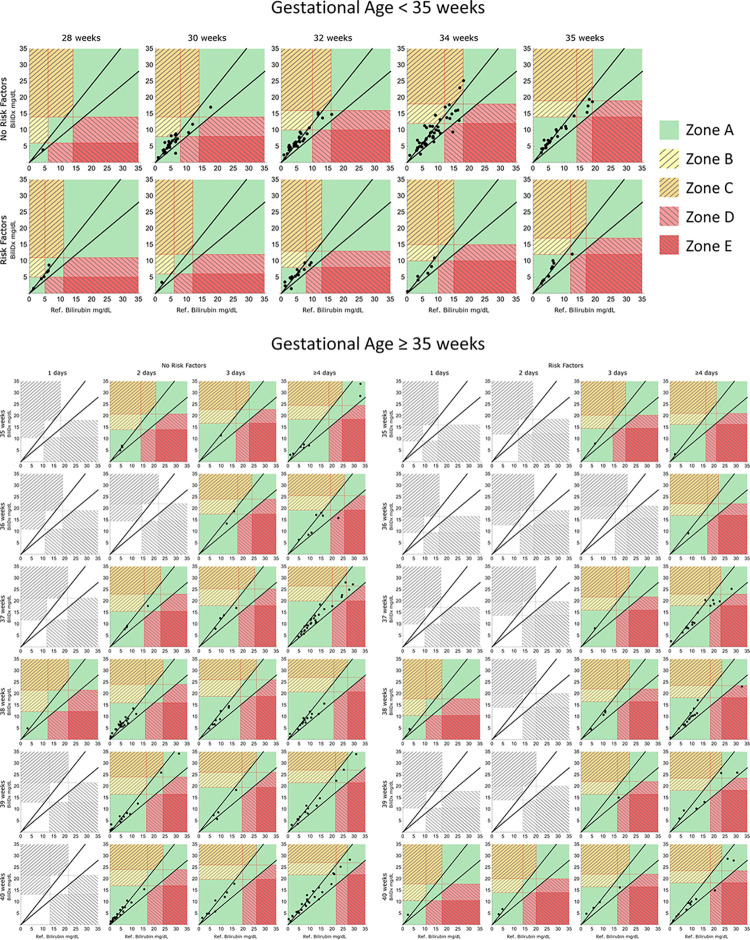
Bilirubin error grids. Treatment thresholds for phototherapy and exchange transfusion (lower & higher red lines, respectively) and CLIA guidelines (black lines) overlaid. Zone A represents correct clinical action; Zones B-E represent increasingly greater potential for harm associated with errors in bilirubin measurement. Colorless grids contain no data points.

**Fig 7:**
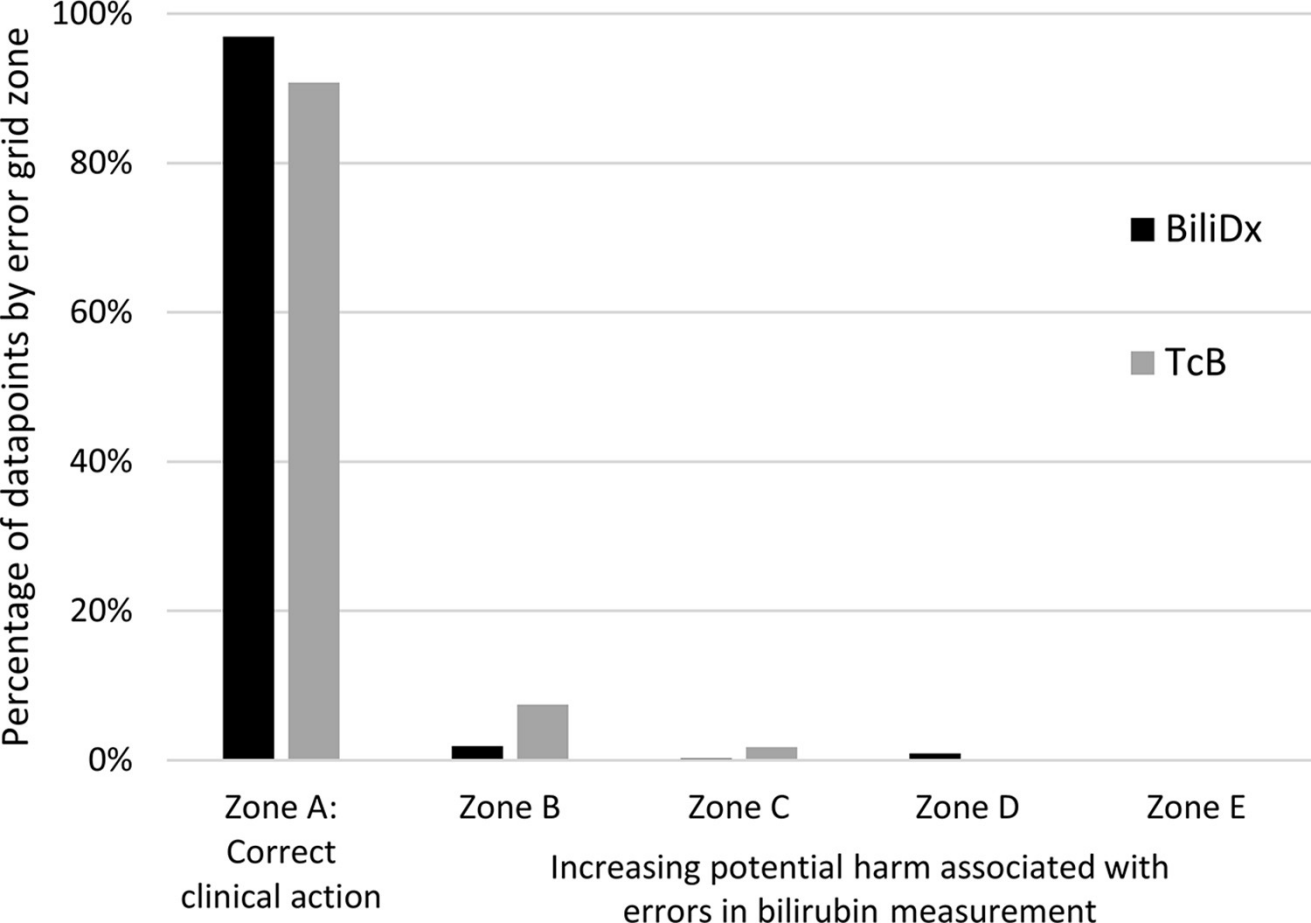
Percentage of samples in the validation set by error grid zone. Zone A represents correct clinical action; Zones B-E represent increasingly greater potential for harm associated with errors in bilirubin measurement. For TcB, 34 samples reporting the non-numeric “>20” were excluded from error grid analysis.

Fig 6 shows error grids documenting the potential clinical impact of BiliDx measurements from the validation set. Of the 446 samples in the validation set, 21 were excluded from this error grid analysis: three because the chronological age of the neonate was less than 24 hours and the guidelines do not cover this age, six were missing both GA and birth weight, and 12 had GA at birth <35 weeks but post-menstrual age at the time of sample collection > = 35 weeks: for these 12 samples, neither set of guidelines applies. One measurement (reference standard TSB level 45.9 mg/dL) is outside the axis limits of Fig 6; this measurement fell into Zone A. Fig 7 summarizes the fraction of measurements in each zone of the error grid; overall, 96.9% of BiliDx measurements fell into Zone A, which represents measurements within CLIA guidelines or that would result in the correct clinical decision. For comparison, 90.8% of TcB measurements fell into Zone A. Additionally, 7.4% of TcB measurements fell into Zone B, which represents overestimation of TSB and potential overtreatment with phototherapy, compared to 1.9% of BiliDx measurements in Zone B.

S2 Fig shows three sets of error grids stratified by direct bilirubin levels (DB) for the 299 BiliDx measurements in the validation set with a DB measurement that meet the inclusion criteria for error grids. All samples meeting the definition of high DB fraction (either DB > 2 mg/dL or DB > 20% of the total bilirubin level, S2 Fig Part C) fell into Zone A, which represents measurements within CLIA guidelines or that would result in the correct clinical decision.

## Discussion

In this work, we evaluated the accuracy of the BiliDx reader and commercially manufactured lateral flow cassettes in two central hospitals in LMICs. Error grid analysis shows that 96.9% of validation set samples measured with BiliDx would have resulted in the same clinical decision as the reference standard. This performance is comparable to previous results with a prototype version of the device that used a handmade two-dimensional paper strip [34].

BiliDx demonstrated comparable accuracy across various HCT levels. In this study, for 1131 samples in which HCT was evaluated, we found an average HCT of 48.8% and range of 17% - 80%. We encountered 69 samples in the validation set with HCT values greater than 60%; BiliDx measurements of these samples showed a mean bias of +1.12 and 95% limits of agreement of -2.70 to 4.93 mg/dL.

BiliDx displays an error message to users when the cassette is not filled with plasma, so the user can either measure the cassette again once plasma completely fills the measurement window or collect another sample and apply it to a new cassette. Overall, only 30 (2.6%) of samples in this study were automatically excluded by BiliDx because the strips did not fill with plasma. One hundred and thirty-three samples (11.8%) had a HCT ≥ 60%; of these samples, 10 (7.5%) were automatically excluded because the cassette did not fill with plasma. By contrast, a 2018 study in Thailand by Thielemans et al. found a 75% error rate for samples with HCT values from 56–65% measured with BiliStick, a similar POC TSB measurement device [23].

BiliDx was also accurate for samples with high levels of direct bilirubin. In this study, we collected 48 samples in the training set and 23 samples in the validation set with high levels of direct bilirubin (DB > 2 mg/dL or > 20% of TSB). BiliDx measurements for the 23 samples in the validation set had a mean bias of -0.74 mg/dL (Fig 5C), indicating that BiliDx underestimated bilirubin levels for these samples. However, the magnitude of the mean bias was still comparable to that of samples with low levels of direct bilirubin (+0.92 mg/dL, Fig 5A), and this magnitude is often not significant enough to affect clinical decision-making, as seen in the error grids. Error grid analysis shows that all 20 samples with high DB levels whose ages allow them to be plotted on an error grid would have resulted in the same clinical decision as the reference standard. This provides strong evidence to support the use of BiliDx to measure bilirubin in samples with high levels of direct bilirubin.

TcB is primarily recommended for screening purposes to evaluate whether TSB measurement is needed [16,45]; when TSB measurement is available, diagnoses and treatment of hyperbilirubinemia should not be based on TcB alone [16,45]. However, in LMICs, it is often used as an alternative to TSB when TSB is not available [8,10,12,18]. In this study, BiliDx demonstrated greater accuracy than TcB; 96.9% of BiliDx measurements in the validation set would have resulted in the same clinical decision as the reference standard, compared to 90.8% of TcB measurements. TcB performance is known to be population-dependent and particularly inaccurate for neonates with dark skin tones [19,46]. We also demonstrated that BiliDx accurately measures TSB levels > 20 mg/dL; in contrast, the Drager JM-105 TcB device displays the message “>20” but does not give a quantitative value above 20 mg/dL. Quantitative assessment of samples with high TSB levels is necessary to determine the need for phototherapy and, potentially, exchange transfusion [36,43].

We developed bilirubin error grids to better understand and visualize the clinical impact of errors in measured bilirubin concentrations. While we also reported the Pearson’s correlation coefficient of BiliDx compared to laboratory standard, this quantity does not give information about the meaningful clinical association between individual TSB measurements in the context of a full clinical picture of each neonate. Thus, bilirubin error grids provide a useful benchmark for evaluating TSB values in the context of a neonate’s medical history, though any clinical decision will likely depend on a clinician’s interpretation and other medical factors. We also note that the updated guidelines used to develop these error grids indicate that they should be used with caution outside of the United States, as the management of hyperbilirubinemia can differ significantly from management in low-resource settings [36].

The strengths of this study include that it was conducted using a commercially manufactured cassette and a prospective algorithm updated to reflect the performance of the new cassette design. This algorithm was evaluated with a larger sample size (1101) and greater bilirubin range to date (0.2–45.9 mg/dL) than those reported in our previous studies [33,34]. We enrolled patients from two different populations at two locations (Lagos, Nigeria and Blantyre, Malawi) during all seasonal variations in temperature and humidity at both locations. We also included both preterm and term neonates, and we included patients who had received phototherapy treatment and/or exchange transfusions. Limitations of this study include that BiliDx was only used by four trained research personnel and only at central hospitals. We also collected a limited number of samples with high DB levels. Finally, our sample size was limited at very high bilirubin concentrations; our dataset included 49 samples with TSB > 20 mg/dL and only 3 samples above 35 mg/dL. Further studies are needed to evaluate BiliDx among a larger number of users and in a greater variety of settings, including primary health centers, as well as with more samples with high TSB and DB levels.

## Conclusion

In conclusion, a low-cost BiliDx reader and commercially manufactured lateral flow cassette performed well in two low-resource central hospitals compared to a reference standard bilirubinometer. BiliDx can accurately measure bilirubin in samples with high HCT and with high DB levels. This evaluation supports the use of BiliDx for accurate, rapid, low-cost, POC TSB measurement in low-resource hospitals.

## Supporting information

S1 Fig BiliDx performance for all measurements of samples in the validation set. Bland-Altman plot for validation set measurements comparing TSB (Total Serum Bilirubin) measured using BiliDx to that measured using a reference standard (UNISTAT). Each sample was measured using one UNISTAT device and either using two BiliDx devices (in the study at LUTH) or using three BiliDx devices (in the study at QECH) for a total of 994 BiliDx measurements. Dashed red lines indicate mean bias; dashed black lines indicate 95% limits of agreement. Diagonal dotted black lines indicate CLIA proposed guidelines of ±20%.
(TIF)

S2 Fig Bilirubin error grids for samples stratified by direct bilirubin levels (DB). Treatment thresholds for phototherapy and exchange transfusion (lower & higher red lines, respectively) and CLIA guidelines (black lines) overlaid. Zone A represents correct clinical action; Zones B-E represent increasingly greater potential for harm associated with errors in bilirubin measurement. A) Samples with DB < 0.2 mg/dL (142 samples). B) Samples with DB between 0.2 and 2 mg/dL, and with DB levels < 20% of the total bilirubin levels (130 samples). C) Samples with either DB > 2 mg/dL or DB > 20% of the total bilirubin level (23 samples).
(TIF)

S1 File (DOCX)

S1 Text Inclusivity in global research. (DOCX)

## Abbreviations

AAP: *American Academy of Pediatrics*
CLIA: *Clinical Laboratory Improvement Amendments*
COMREC: *College of Medicine Research Ethics Committee*
DB: *Direct Bilirubin*
GA: *Gestational age (weeks)*
HCT: *Hematocrit (%)*
IRB: *Institutional Review Board*
LMICs: *Low and middle-income countries*
LUTH: *Lagos University Teaching Hospital*
OD: *Optical density (unitless)*
POC: *point of care*
QECH: *Queen Elizabeth Central Hospital*
TSB: *Total serum bilirubin concentration (mg/dL)*
TcB: *Transcutaneous bilirubin concentration (mg/dL)*

## References

[1] Olusanya BO, Teeple S, Kassebaum NJ. The contribution of neonatal jaundice to global child mortality: Findings from the GBD 2016 Study. Pediatrics. 2018;141(2). doi: 10.1542/peds.2017-1471 29305393

[2] Keren R, Tremont K, Luan X, Cnaan A. Visual assessment of jaundice in term and late preterm infants. Arch Dis Child Fetal Neonatal Ed [Internet]. 2009 Sep 1;94(5):F317–22. Available from: http://fn.bmj.com/content/94/5/F317.abstract. doi: 10.1136/adc.2008.150714 19307221

[3] Bhutani VK, Stark AR, Lazzeroni LC, Poland R, Gourley GR, Kazmierczak S, et al. Predischarge screening for severe neonatal hyperbilirubinemia identifies infants who need phototherapy. Journal of Pediatrics [Internet]. 2013;162(3):477–482.e1. Available from: doi: 10.1016/j.jpeds.2012.08.022 23043681

[4] Bhutani VK, Zipursky A, Blencowe H, Khanna R, Sgro M, Ebbesen F, et al. Neonatal hyperbilirubinemia and Rhesus disease of the newborn: incidence and impairment estimates for 2010 at regional and global levels. Pediatr Res. 2013;74(December):86–100. doi: 10.1038/pr.2013.208 24366465 PMC3873706

[5] Sarici SU, Serdar M, Korkmaz A, Erdem G, Oran O, Tekinalp G, et al. Incidence, Course, and Prediction of Hyperbilirubinemia in Near-Term and Term Newborns. Pediatrics. 2004;113(4):775–80. doi: 10.1542/peds.113.4.775 15060227

[6] Watchko JF, Tiribelli C. Bilirubin-Induced Neurologic Damage—Mechanisms and Management Approaches. New England Journal of Medicine. 2013;369(21):2021–30. doi: 10.1056/NEJMra1308124 24256380

[7] Arnolda G, Chien TD, Hayen A, Hoi NTX, Maningas K, Joe P, et al. A comparison of the effectiveness of three LED phototherapy machines, single- and double-sided, for treating neonatal jaundice in a low resource setting. PLoS One. 2018;13(10):1–12. 10.1371/journal.pone.0205432 PMC6181361 30308024

[8] Olusanya BO, Kaplan M, Hansen TWR. Neonatal hyperbilirubinaemia: a global perspective. Lancet Child Adolesc Health. 2018;2(8):610–20. doi: 10.1016/S2352-4642(18)30139-1 30119720

[9] Hulzebos C V., Vitek L, Coda Zabetta CD, Dvořák A, Schenk P, van der Hagen EAEE, et al. Diagnostic methods for neonatal hyperbilirubinemia: benefits, limitations, requirements, and novel developments. Pediatr Res. 2021 May;(April). 10.1038/s41390-021-01546-y 33948000

[10] O’Hare B, Kawaza K, Mzikamanda R, Molynuex L. Care of the Infant and Newborn (COIN 2017) Participants Manual. 2017; Available from: https://risweb.st-andrews.ac.uk/portal/en/researchoutput/care-of-the-infant-and-newborn-in-malawi-2017(9481917d-668f-4cf6-aa5c-e8d54689a359).html.

[11] Kramer L. Advancement of Dermal Icterus in the Jaundiced Newborn. The American Journal of Diseases of Children. 1969;118(3):454–8. doi: 10.1001/archpedi.1969.02100040456007 5817480

[12] Olusanya BO, Ogunlesi TA, Kumar P, Boo NYY, Iskander IF, de Almeida MFB, et al. Management of late-preterm and term infants with hyperbilirubinaemia in resource-constrained settings. BMC Pediatr. 2015;15(1):1–12. 25884679 10.1186/s12887-015-0358-z PMC4409776

[13] Moyer VA, Ahn C, Sneed S. Accuracy of clinical judgment in neonatal jaundice. Arch Pediatr Adolesc Med. 2000;154(4):391–4. doi: 10.1001/archpedi.154.4.391 10768679

[14] National Institute for Health and Care Excellence. Jaundice in newborn babies under 28 days [Internet]. NICE guideline. 2010 [cited 2022 Aug 9]. p. 1–25. Available from: https://www.nice.org.uk/guidance/cg98.

[15] El-Beshbishi SN, Shattuck KE, Mohammad AA, Petersen JR. Hyperbilirubinemia and transcutaneous bilirubinometry. Clin Chem. 2009;55(7):1280–7. doi: 10.1373/clinchem.2008.121889 19443565

[16] Maisels MJ, Bhutani VK, Bogen D, Newman TB, Stark A, Watchko J. Hyperbilirubinemia in the Newborn Infant ≥35 Weeks’ Gestation: An Update with Clarifications. Pediatrics. 2009;124(4):1193–8. 19786452 10.1542/peds.2009-0329

[17] Drager. Sample Usage Protocol: Jaundice Meter JM-105 [Internet]. 2018 [cited 2022 Aug 8]. Available from: https://www.draeger.com/Library/Content/jaundice-meter-sample-usage-protocol-template-MU25261-en.pdf.

[18] Rylance S, Yan J, Molyneux E. Can transcutaneous bilirubinometry safely guide phototherapy treatment of neonatal jaundice in Malawi? Paediatr Int Child Health. 2014;34(2):101–7. doi: 10.1179/2046905513Y.0000000050 24090969

[19] Olusanya BO, Imosemi DO, Emokpae AA. Differences between transcutaneous and serum bilirubin measurements in black African neonates. Pediatrics. 2016 Sep 1;138(3):e20160907–e20160907. doi: 10.1542/peds.2016-0907 27577578

[20] Maya-Enero S, Candel-Pau J, Garcia-Garcia J, Duran-Jordà X, López-Vílchez MÁ. Reliability of transcutaneous bilirubin determination based on skin color determined by a neonatal skin color scale of our own. Eur J Pediatr. 2021;180:607–16. doi: 10.1007/s00431-020-03885-0 33409587

[21] Varughese PM, Krishnan L, Ravichandran. Does Color Really Matter? Reliability of Transcutaneous Bilirubinometry in Different Skin-Colored Babies. Indian Journal of Paediatric Dermatology. 2018;19(4):315–20.

[22] Jacob EA. Hematological Differences in Newborn and Aging: A Review Study. Hematology & Transfusion International Journal. 2016;3(3):178–90.

[23] Thielemans L, Hashmi A, Priscilla DD, Kho Paw M, Pimolsorntong T, Ngerseng T, et al. Laboratory validation and field usability assessment of a point-of-care test for serum bilirubin levels in neonates in a tropical setting. Wellcome Open Res [Internet]. 2018;3(110):1–19. Available from: https://wellcomeopenresearch.org/articles/3-110/v1. 30271889 10.12688/wellcomeopenres.14767.1 PMC6137410

[24] Greco C, Iskander IF, El Houchi SZ, Rohsiswatmo R, Rundjan L, Ogala WN, et al. Diagnostic Performance Analysis of the Point-of-Care Bilistick System in Identifying Severe Neonatal Hyperbilirubinemia by a Multi-Country Approach. EClinicalMedicine. 2018;1:14–20. doi: 10.1016/j.eclinm.2018.06.003 31193593 PMC6537563

[25] Calmark. Calmark POC Test: Neo-Bilirubin, Fast detection of bilirubin in newborns [Internet]. [cited 2021 Apr 22]. Available from: https://assets.website-files.com/5a6f1d11f5d2f1000178a4a9/5fa4019c8235fd515b7590b1_PL_Calmark_Neo-Bilirubin_EN_v201.pdf.

[26] Bilimetrix. BiliStick System Technical Datasheet [Internet]. 2016 [cited 2021 Apr 22]. p. 1–3. Available from: https://www.bilimetrix.net/wp-content/themes/hypnotherapy-child/pdf/Bilistick_SystemTechnical_DataSheet_EN.pdf

[27] Coda Zabetta CD, Iskander IF, Greco C, Bellarosa C, Demarini S, Tiribelli C, et al. Bilistick: A low-cost point-of-care system to measure total plasma bilirubin. Neonatology. 2013 Jan;103(3):177–81. doi: 10.1159/000345425 23295342

[28] Lee ACC, Folger L V., Rahman M, Ahmed S, Bably NN, Schaeffer L, et al. A novel icterometer for hyperbilirubinemia screening in low-resource settings. Pediatrics. 2019;143(5). doi: 10.1542/peds.2018-2039 30952779

[29] Olusanya BO, Slusher TM, Imosemi DO, Emokpae AA. Maternal detection of neonatal jaundice during birth hospitalization using a novel two-color icterometer. PLoS One. 2017;12(8). doi: 10.1371/journal.pone.0183882 28837635 PMC5570328

[30] Thompson BL, Wyckoff SL, Haverstick DM, Landers JP. Simple, Reagentless Quantification of Total Bilirubin in Blood Via Microfluidic Phototreatment and Image Analysis. Anal Chem. 2017;89(5):3228–34. doi: 10.1021/acs.analchem.7b00354 28192917

[31] Bell JG, Mousavi MPS, Abd El-Rahman MK, Tan EKW, Homer-Vanniasinkam S, Whitesides GM. Paper-based potentiometric sensing of free bilirubin in blood serum. Biosens Bioelectron. 2019;126(August 2018):115–21. doi: 10.1016/j.bios.2018.10.055 30396018

[32] Tan W, Zhang L, Doery JCG, Shen W. Three-dimensional microfluidic tape-paper-based sensing device for blood total bilirubin measurement in jaundiced neonates. Lab Chip. 2020;20(2):394–404. doi: 10.1039/c9lc00939f 31853529

[33] Keahey PA, Simeral ML, Schroder KJ, Bond MM, Mtenthaonnga PJ, Miros RH, et al. Point-of-care device to diagnose and monitor neonatal jaundice in low-resource settings. Proceedings of the National Academy of Sciences. 2017;114. doi: 10.1073/pnas.1714020114 29203650 PMC5754796

[34] Shapiro A, Anderson J, Mtenthaonga P, Kumwenda W, Bond M, Schwarz R, et al. Evaluation of a Point-of-Care Test for Bilirubin in Malawi. Pediatrics. 2022;150(2). doi: 10.1542/peds.2021-053928 35799070

[35] Hertz H, Dybkaer R. Molar Absorption Coefficients for Bilirubins in Adult and Infant Serum with Determination of an Isosbestic Point. Scand J Clin Lab Invest. 1972;29(2):217–30. doi: 10.3109/00365517209081079 4555160

[36] Kemper AR, Newman TB, Slaughter JL, Jeffrey Maisels M, BCh M, Watchko JF, et al. CLINICAL PRACTICE GUIDELINE Guidance for the Clinician in Rendering Pediatric Care Clinical Practice Guideline Revision: Management of Hyperbilirubinemia in the Newborn Infant 35 or More Weeks of Gestation FROM THE AMERICAN ACADEMY OF PEDIATRICS [Internet]. Vol. 150, Pediatrics. 2022. Available from: http://publications.aap.org/pediatrics/article-pdf/150/3/e2022058859/1375979/peds_2022058859.pdf. 10.1542/peds.2022-058859 35927462

[37] Bond M, Mvula J, Molyneux E, Richards-Kortum R. Design and performance of a low-cost, handheld reader for diagnosing anemia in Blantyre, Malawi. IEEE Conference Proceedings. 2014;(0940902):267–70. 10.1109/HIC.2014.7038926 PMC4409007 25918750

[38] Pendse A, Jasani B, Nanavati R, Kabra N. Comparison of transcutaneous bilirubin measurement with total serum bilirubin levels in preterm neonates receiving phototherapy. Indian Pediatr. 2017;54(8):641–3. doi: 10.1007/s13312-017-1126-y 28891475

[39] Harb R, Thomas D. Conjugated Hyperbilirubinemia: Screening and Treatment in Older Infants and Children. Gastroenterology. 2007;28(3):83–91. doi: 10.1542/pir.28-3-83 17332167

[40] Pan DH, Rivas Y. Jaundice: Newborn to age 2 months. Pediatr Rev. 2017;38(11):499–510. doi: 10.1542/pir.2015-0132 29093118

[41] Dell RB, Ramakrishnan R. Sample Size Determination. ILAR J. 2002;43:207–13. doi: 10.1093/ilar.43.4.207 12391396 PMC3275906

[42] Clarke WL, Cox D, Gonder-Frederick L, Carter William, Pohl SL. Evaluating clinical accuracy of systems for self-monitoring of blood glucose. Diabetes Care. 1987;10(5):622–8. 3677983 10.2337/diacare.10.5.622

[43] Maisels MJ, Watchko JF, Bhutani VK, Stevenson DK. An approach to the management of hyperbilirubinemia in the preterm infant less than 35 weeks of gestation. Journal of Perinatology. 2012;32(9):660–4. doi: 10.1038/jp.2012.71 22678141

[44] Westgard JO, Westgard S. New CLIA proposed rules for acceptance limits for proficiency testing [Internet]. Westgard QC. 2019 [cited 2021 Apr 22]. Available from: https://www.westgard.com/ 2019-clia-changes.htm.

[45] Bosschaart N, Kok JH, Newsum AM, Ouweneel DM, Mentink R, Van Leeuwen TG, et al. Limitations and opportunities of transcutaneous bilirubin measurements. Pediatrics. 2012;129(4):689–94. doi: 10.1542/peds.2011-2586 22430456

[46] Maisels JM, Ostrea EM, Touch S, Clune S, Cepeda E, Kring E, et al. Evaluation of a New Transcutaneous Bilirubinometer. Pediatrics. 2004;113(6):1628–35. doi: 10.1542/peds.113.6.1628 15173483

