# Supplementary figures and images for: Design and field evaluation of a lateral flow cassette device for point-of-care bilirubin measurement
Source: PLOS Glob Public Health. 2023 Aug 8;3(8):e0002262. doi: 10.1371/journal.pgph.0002262 (PMC10409260; doi:10.1371/journal.pgph.0002262)

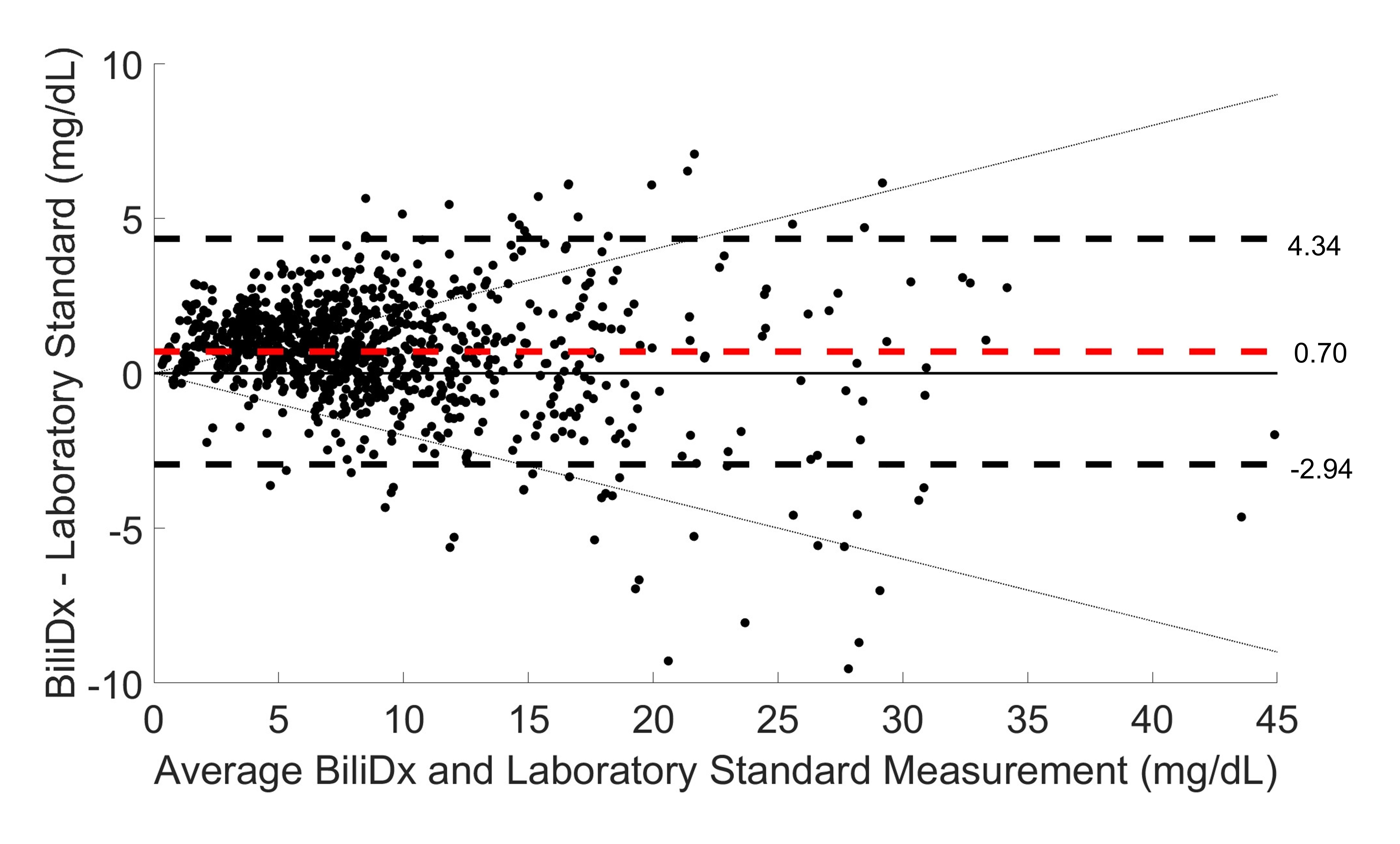

Supplement: S1 Fig — Bland-Altman plot for validation set measurements comparing TSB (Total Serum Bilirubin) measured using BiliDx to that measured using a reference standard (UNISTAT). Each sample was measured using one UNISTAT device and either using two BiliDx devices (in the study at LUTH) or using three BiliDx devices (in the study at QECH) for a total of 994 BiliDx measurements. Dashed red lines indicate mean bias; dashed black lines indicate 95% limits of agreement. Diagonal dotted black lines indicate CLIA proposed guidelines of ±20%. (TIF) [file pgph.0002262.s001.TIF]

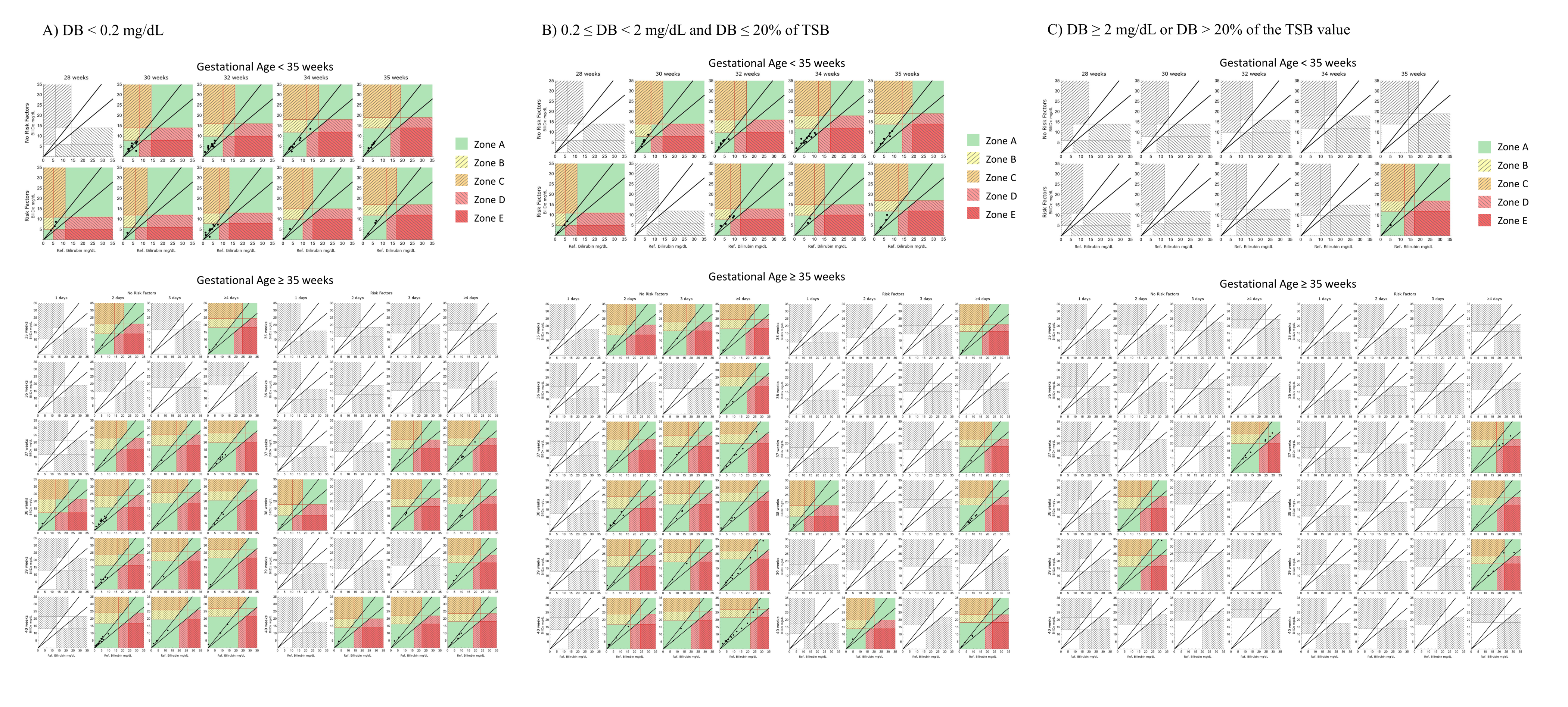

Supplement: S2 Fig — Treatment thresholds for phototherapy and exchange transfusion (lower & higher red lines, respectively) and CLIA guidelines (black lines) overlaid. Zone A represents correct clinical action; Zones B-E represent increasingly greater potential for harm associated with errors in bilirubin measurement. A) Samples with DB < 0.2 mg/dL (142 samples). B) Samples with DB between 0.2 and 2 mg/dL, and with DB levels < 20% of the total bilirubin levels (130 samples). C) Samples with either DB > 2 mg/dL or DB > 20% of the total bilirubin level (23 samples). (TIF) [file pgph.0002262.s002.TIF]
