# Supplementary material for: Design and field evaluation of a lateral flow cassette device for point-of-care bilirubin measurement
Source: PLOS Glob Public Health. 2023 Aug 8;3(8):e0002262. doi: 10.1371/journal.pgph.0002262 (PMC10409260; doi:10.1371/journal.pgph.0002262)
Supplement: S1 File — (DOCX) [file pgph.0002262.s003.docx]

Caption:

BiliDx cassette for point-of-care bilirubin measurement. A lateral flow strip is enclosed in an injection molded plastic cassette. A heel stick is performed on a neonate, and capillary blood is collected into a 75 µL plastic transfer pipette. Blood is applied to the sample port of the BiliDx cassette. Image credit: Shapiro et al.

We confirm that we are the photographers of the striking image. We confirm that the image may be published under the Creative Commons Attribution License.
